# 

**Published:** 1899-09

**Authors:** 


### CONTENTS.

ORIGINAL ARTICLES:
- Army Veterinary Service. By RUSH S. S. HUIDEKOPER, M.D. . . . . . 537
- On the Importance of Keeping a Case-book and Reporting Cases. By JOHN J. REPP, V.M.D. . . . . . . . . . . 547
- Azoturia. By RICHARD P. LYMAN, M D.V. 552
- Cotton-seed Disease. Dy Dr. F. C. McCURDY 560
- Formalin as a Surgical Dressing. By CHARLES H. HIGGINS, B.S., D.V.S. . . 567
- Tuberculosis. By V. G. HUNT, V.S. . . 569

SELECTION:
- A Contribution to the Study of the Infectiousness of Milk from Tuberculous Cows and as to the Value of the Tuberculin-test. By Dr. LYDIA RABINOWITCH and Dr. WALTER KEMPNER . . . . . 572

THERAPEUTICAL AND PHARMACEUTICAL NOTES
- Constituents of Digitalis Leaves—Medicaments which Age Affects—Drugs which Should Not be Dispensed in Capsule Form—Decomposition of Chloroform—Fluid Extract of Hyoscyamus—Tenaline—Law's Sheep Dip—Pancreatic Secretion—Kaiser's Sheep Dip—Grafts of Living Bone—Dog Soap—Preparation of Tincture of Iodine—Cerates of Cantharides—Compound Tincture of Opium—Tincture of Chloride of Iron—Scab in Sheep—To Protect Cattle from Flies—Camphor as an Antidote to Carbolic Acid—Deadly "Headache Powders" — Pyoktanin: Methyl-violet—Solution for Eczema . 582

DEPARTMENT OF SURGERY:
- Trephining: Its Indications, Technique, After treatment, and Sequelæ. By L. A. MERRILAT, V.S. . . . . . . . 587
- A New Method of Castrating Horses . 589
- Advantages of Aseptic Surgery . . . 590

EDITORIAL:
- Another Veterinary Journal—Another Veterinary College . . . . . . . 591

COMMUNICATION . . . . . . . . 592

AMONG THE COLLEGES . . . . . . 593

SOCIETY PROCEEDINGS:
- Chicago — Pennsylvania — Report of the Committee on Army Legislation . . 594

PERSONALS . . . . . . . . . 600

